# Interaction of sperm with endometrium can regulate genes involved in endometrial receptivity pathway in mice: An experimental study

**DOI:** 10.18502/ijrm.v13i10.7765

**Published:** 2020-10-13

**Authors:** Marziyeh Ajdary, Zahra Zandieh, Fatemeh Sadat Amjadi, Fariborz Keyhanfar, Mehdi Mehdizadeh, Reza Aflatoonian

**Affiliations:** ^1^Endometriosis Research Center, Iran University of Medical Sciences, Tehran, Iran.; ^2^Cellular and Molecular Research Center, Iran University of Medical Sciences, Tehran, Iran.; ^3^Department of Anatomical Science, Iran University of Medical Sciences, Tehran, Iran.; ^4^Department of Pharmacology, School of Medicine, Iran University of Medical Sciences, Tehran, Iran.; ^5^Department of Endocrinology and Female Infertility, Reproductive Biomedicine Research Center, Royan Institute for Reproductive Biomedicine, ACECR, Tehran, Iran.

**Keywords:** Endometrial receptivity, Gene expression, Mice

## Abstract

**Background:**

Many researchers consider implantation and endometrial receptivity as pertinent issues in reproductive science. Although, several experiments have been performed and their results evaluated, yet there is no confirmed evidence about the related factors and the role of sperm in endometrial receptivity.

**Objective:**

To investigate the effect of the sperm-endometrium interaction in regulating genes involved in the endometrial receptivity pathway.

**Materials and Methods:**

In this experimental study, 10 male and 30 female NMRI mice were included, and half of the male cases were vasectomized. The subjects were divided into two groups as follows; group 1 (case) comprised of 15 females mated with 5 non-vasectomized male mice, while group 2 (control) consisted of 15 females mated with 5 vasectomized males. Cases were sacrificed and assessed after 36 hr and the endometrial tissue was extracted and kept at -80°C until the next use. The expression of the endometrial receptivity pathway genes, including *VEGF, HBEGF, FGF2, EGF, LIF, LIFR, HOXA10, MUC1, PGR, *and *CSF,* was examined in both groups. For statistical analysis, an independent samples test (Mean ± SD) was used.

**Results:**

The mRNA levels of *LIF* (p = 0.045), *LIFR* (p = 0.040), *MUC1* (p = 0.032), *VEGF *(p = 0.022), *EFG *(p = 0.035), *and FGF2* (p = 0.040) were significantly upregulated in the case group compared with the control group.

**Conclusion:**

Finally, seminal plasma was observed to be effective in expressing the involved genes in the successful implantation pathway, including *LIF, LIFR, MUC1, VEGF, EGF, *and *FGF2*.

## 1. Introduction

While several studies have been performed to provide potential molecules, candidates for endometrial receptivity (1-5), there is yet no confirmed evidence about these molecules and the role of sperm in endometrial receptivity. During transportation, fertilization, and development, spermatozoa interact with the endometrial cells and create a category of secretory molecules (6), influencing the endometrial receptivity (7).

In a study, Berger and colleague demonstrated that leukemia inhibitory factor (*LIF*), Leukemia inhibitory factor receptor *(LIFR)*, Homeobox gene 10 (*HOXA10*), Mucin 1 (*MUC1)*, Progesterone receptor (*PGR)*, Colony Stimulating Factor *(CSF)*, Vascular endothelial growth factor *(VEGF)*, Heparin-binding EGF-like growth factor (*HBEGF)*, Epidermal growth factor *(EGF),* and Fibroblast Growth Factor 2 *(FGF2) *are involved in endometrial receptivity (8). In a study of mice and pigs, Robertson and colleague found that the seminal plasma is effective on the cytokines and epithelial cocaine cells of the uterus, which makes it successful in implantation (9). Several hormones and cytokines that play a role in endometrial receptivity have been established by studies. *HOXA10*, *LIF* are involved in the proliferation, differentiation, and decidualization of the endometrium and are highly expressed at this phase (10). While the *LIF-R* gene plays a role in mediating the action of the leukemia-inhibitory factor that finally causes a proliferation (11), *HB-EGF* plays an endometrial preparation for embryo acceptance and is expressed in mice on the fourth day of gestation (12). *VEGF, FGF-2, EGF* are involved in angiogenesis that is important at the time of implantation and transportation of nutrients and oxygen to the embryo (13). *MUC1* at the surface of uterine epithelia, acts as a barrier to microbial infection and enzymatic attack. At the time of implantation, the expression of *MUC1* reduce *PGR* and *PGR *and *MUC1* have different expression during a natural cycle in mice. *MUC1* and *PGR* are upregulated at estrous and at early pregnancy for four days and reduced on day 4 of the pregnancy (14). Additionally, *CSF* plays an important role in the proliferation and differentiation, and starting from the third day of gestation, its expression increases (15).

In this study, the effects of sperm interaction with endometrium on endometrial receptivity gene expression (*LIF*, *LIFR*, *HOXA10*, *MUC1*, *PGR*, *CSF*, *VEGF*, *HBEGF*, *EGF*, *and FGF2*) in a mouse model (*in-vivo) *were investigated. All of these genes fall in the path of implantation and affect the endometrial readiness in embryo acceptance. Because studies have shown that both spermatozoa and semen are effective on pinopods (1, 16), in this study, we sought to investigate the effect of sperm on implantation and the genes important for uterine reception.

## 2. Materials and Methods

### Materials and equipment

Entire objective includes Ketamine (Alfasan, Woerden- Holland), Xylazine (Alfasan, Woerden-Holland), TRIzol reagent (Sigma, Pool, UK), cDNA Synthesis Kit (Invitrogen, Paisley, UK), primers (Roche, Basel, Switzerland), and SYBR Green PCR Master Mix (Invitrogen, Paisley, UK). Entire Apparatus includes an inverted microscope (Nikon UK, Ltd.), Nanodrop ND-100 spectrophotometer (Thermo Fisher Scientific, USA), thermocycler-T100 (Bio-Rad, USA), Opticon II system (Invitrogen, Paisley, UK), Step One Plus Real-time PCR (Applied Biosystems, Foster, USA).

### Animal preparation

In this experimental study, 30 female and 10 male NMRI mice weighing 20-50 gr at 8 wk of age were used. Half of the male animals were subjected to vasectomy and anesthetized with Ketamine (10 mg/kg) or Xylazine (2%). One month after wound healing, the mice were selected to continue the experiments.

1. Fifteen female mice were mated with the vasectomized male mice. Two days later, they were coupled with a sexually mature male mouse at 05:00 in the afternoon. The next morning, the vaginal plugs were examined, and therefore, this day was named the first gestation day. Female mice were sacrificed with perfusion-fixation technique on day 1.5 post coitus. The uterus was removed and the endometrial tissues were collected and frozen in liquid nitrogen and stored at -80°C for examination by quantitative real-time PCR (Q-PCR).

2. Fifteen female mice were mated with the non-vasectomized male mice. Two days later, they were coupled with a sexually mature male mouse at 05:00 in the afternoon two days later. The next morning, the vaginal plugs were examined and therefore, this day was named the first gestation day. The female mice were then sacrificed with the perfusion-fixation technique on day 1.5 post copulation. The endometrial tissue on day 1.5 was washed by PBS and sperms present in the uterus were observed under a microscope (1).

The uterus was removed, washed, and the endometrial tissues were collected and frozen in liquid nitrogen and stored at -80°C for examination under Q-PCR.

### RNA extraction and real-time quantitative reverse-transcription PCR

Extraction and purification of the total RNAs from the endometrial tissue were performed by TRIzol reagent (Sigma, Pool, UK) based on the given instructions. A Nanodrop ND-100 spectrophotometer was applied to determine the exact concentration of RNA. In order to evaluate the level of gene expression, a Q*-*PCR was used against *HBEGF, LIF, PGR, FGF2, VEGF, HOXA10, LIFR, CSF, EGF, *and *MUC1*. A transcript or High-Fidelity cDNA Synthesis Kit (Invitrogen, Paisley, UK) was used for the reverse transcription of a 500-ng sample of RNA using oligo (dT) primers (Roche, Basel, Switzerland). In order to amplify the cDNA (1 μL), the SYBR Green PCR Master Mix (Invitrogen, Paisley, UK) and the Opticon II system (Invitrogen, Paisley, UK) were used according to the instructions. The annealing temperature of 60°C for all tested genes were done to find the 40 PCR cycles. Further, primers were specifically designed between two adjacent exons (Gene Runner program). Table I presents all the used sequences. The concentrations of mRNA for these genes (Ct) were regulated via the reference gene glyceraldehyde-3-phosphate dehydrogenase (β*-actin*) through subtracting the Ct value of β*-actin* from the Ct value of the sample (ΔCt = Ct${}_{\mathrm{Sample}}$- Ct${}_{\mathrm{Reference}}$). 2-ΔΔCt was applied for quantification of the relative expression of the target gene to the calibrator (17, 18).

Additionally, the Primer Premier 5.0 software was used to design primer pairs specified for each gene. The applied primers for real-time PCR are presented in Table I.

**Table 1:** The sequence of the primers used in the current study


**Gene**	**Sequence (5'->3')**	**Length**	**Tm**	**GC%**	**Self-complementarity**	**Self-3' complementarity**
*MUC1*	Forward primer	TAGCATCAAGTTCAGGTCAGGC	22	60.36	50.00	3.00	2.00
	Reverse primer	GACTTCACGTCAGAGGCACTAA	22	60.03	50.00	5.00	3.00
*EGF*	Forward primer	ACTGGACGGTTTGCCTCTTT	20	59.82	50.00	3.00	0.00
	Reverse primer	ATTCAGGGGTTGACAGAGCAT	21	59.36	47.62	3.00	2.00
*HBEGF*	Forward primer	CCCAGAAGAGATTGAGCATCCA	22	59.83	50.00	3.00	2.00
	Reverse primer	ACCCGAAGAACAGCAGGATAAG	22	60.09	50.00	2.00	0.00
*FGF2*	Forward primer	GACCCACACGTCAAACTACAAC	22	59.71	50.00	4.00	0.00
	Reverse primer	CTGTAACACACTTAGAAGCCAGC	23	59.57	47.83	5.00	2.00
*HOXA10*	Forward primer	TGTTTAATCAGGGAGTCCAGGC	22	60.03	50.00	6.00	2.00
	Reverse primer	TTTTTCAACCAGCCAGGTCAAG	22	59.57	45.45	5.00	1.00
*CSF1*	Forward primer	GGCATCATCCTAGTCTTGCTGA	22	59.90	50.00	4.00	3.00
	Reverse primer	AATCCAATGTCTGAGGGTCTCG	22	59.83	50.00	3.00	2.00
*LIF*	Forward primer	TTTCCAGGTACTCACTGCACTC	22	59.96	50.00	4.00	2.00
	Reverse primer	TCTCAGACCAACACCCTCATTG	22	59.96	50.00	5.00	3.00
*LIFR*	Forward primer	TGTCTGCTGACTTCTTCACCTC	22	59.96	50.00	6.00	0.00
	Reverse primer	TAACACGAGTGCTACTGGTTCC	22	60.03	50.00	6.00	2.00
*PGR*	Forward primer	AAAACTGCCCAGCATGTCGT	20	60.82	50.00	4.00	1.00
	Reverse primer	CAACACCGTCAAGGGTTCTCAT	22	60.81	50.00	5.00	2.00
*VEGFA*	Forward primer	TGCAGATTATGCGGATCAAACC	22	59.38	45.45	5.00	2.00
	Reverse primer	TGCATTCACATTTGTTGTGCTGTAG	22	61.02	40.00	4.00	2.00
*β-actin*	Forward primer	CAAGATCATTGCTCCTCCTG	20	58.4	50.00	6.00	1.00
	Reverse primer	ATCCACATCTGCTGGAAGG	19	57.3	52.60	6.00	0.00

### Ethical consideration

All experiments were done following the National Institutes of Health Guide for the Care and Use of Laboratory Animals (Iran University of Medical Sciences) and were approved by the Research and Ethics Committee of Iran University of Medical Sciences (code: IR-IUMS.1394.94-05-117-27524).

### Statistical Analysis

All groups were analyzed using an independent sample *t* test. Statistical analyses were performed using the SPSS (version 21.0, IBM, New York, USA) software. The results were considered significant at p < 0.05 and are expressed as Mean ± SD.

## 3. Results

### Gene expression in endometrial tissue

**Figure 1:**
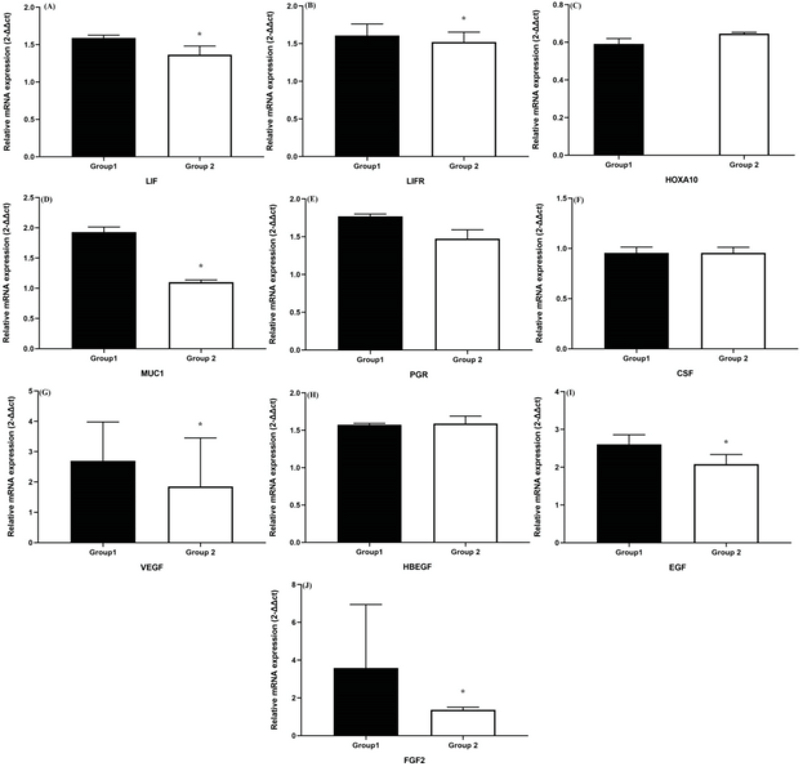
Graphs showing the mean mRNA levels of experimental groups. Group 1 indicates females mated with non-vasectomized male mice, while Group 2 indicates those mated with vasectomized male mice. Data are shown as mean ± standard error of the mean (SD; n = 10 each group). Β-actin was used as an inner control. (A) *LIF*, (B) *LIFR*, (C) *HOXA10*, (D) *MUC1*, (E) *PGR*, (F) *CSF*, (G) *VEGF*, (H) *HBEGF*, (I) *EGF*, (J) *FGF2*. *< 0.05. The comparison was made between groups 1 and 2.

The mRNA levels of *LIF, MUC1, VEGF, EFG *and *FGF2* were all significantly higher in the group that was mated with the non-vasectomized males compared with the group mated with the vasectomized males (p < 0.05) (Figure 1, Table II).

No significant differences were found in the expression of *PGR*, *CSF*, *HBEGF*, and *HOXA10* mRNA between the group that was mated with non-vasectomized males and the group that was mated with vasectomized males; however, p > 0.05 was observed (Figure 1, Table II).

The *LIF*, *LIFR*, *MUC1*, and *FGF2* were mainly expressed in the cytoplasm of both the endometrial luminal epithelial cells and the glandular epithelial cells (Figure 1). As shown in Figure 1, the expression levels of *LIF*, *LIFR*, *MUC1*, and *FGF2* in the group that was mated with the non-vasectomized males were higher compared with the group that was mated with the vasectomized males (all p < 0.05). As shown in Figure 1, the expression levels of *HOXA10* in the group that was mated with the non-vasectomized males were lower compared with the group that was mated with the vasectomized males.

**Table 2:** The mean mRNA levels of experimental groups


**Gene **	**Groups**	**mRNA levels**	**P-value**
*LIF*	Group1	1.5901 ± 0.03731	
	Group2	1.3659 ± 0.11553	0.045
*LIFR*	Group1	1.6069 ± 0.15460		0.040
	Group2	1.5209 ± 0.13303		
*MUC1*	Group1	1.9303 ± 0.08292	0.032
	Group2	1.1007 ± 0.03772	
*VEGFA*	Group1	2.6990 ± 1.28129	0.022
	Group2	1.8494 ± 1.60188	
*EGF*	Group1	2.6082 ± 0.25102	0.035
	Group2	2.0843 ± 0.25288	
*FGF2*	Group1	3.5839 ± 3.35855	0.040
	Group2	1.3650 ± 0.14588	
*PGR*	Group1	1.7696 ± 0.03102	0.059
	Group2	1.4751 ± 0.11781	
*CSF*	Group1	0.9569 ± 0.05692	0.559
	Group2	0.9564 ± 0.05570	
*HBEGF*	Group1	1.5736 ± 0.02025	0.921
	Group2	1.5907 ± 0.10027	
*HOXA10*	Group1	0.5920 ± 0.02809	0.130
	Group2	0.6460 ± 0.00737	
Groups were analyzed using independent sample *t* test, Data presented as Mean ± SD, Group 1 comprised of females mated with non-vasectomized male mice and Group 2 (control) females mated with vasectomized male			

## 4. Discussion

The results of this study confirmed that seminal plasma regulates the *LIF*, *LIFR*, *HOXA10*, *MUC1*, *PGR*, *CSF*, *VEGF*, *HBEGF*, *EGF*, and *FGF2* expression in the mouse endometrium in vivo. According to our results, it can be recommended that seminal plasma, by changing the gene expression, can regulate the environment within the endometrium.

During the interaction of seminal plasma-endometrium, genes involved in proliferation, differentiation, and decidualization are expressed by the epithelial cell, and endometrial cells secrete inflammatory factors, growth factor, cytokines, including *LIF*, *VEGF*, *FGF2*, and *EGF *(19-21). The *VEGF*, *FGF2*, and *EGF* increase endometrial angiogenesis and subsequently lead to a successful implantation (22). In addition, the expression of the *HBEGF* factor at this stage prepares the uterus for endometrial receptivity (23). During the interaction of the seminal plasma-endometrium, the expression of *HBEGF*, *CSF, PGR* increases, which plays an important role in the proliferation and differentiation (15). At one stage of estrus (the time of mating of the animal), the innate immune factors in the uterus of the female animal increase; one of the most important of these factors is the *MUC1* gene, which prevents microbes from entering the uterus (24).

Intriguingly, numerous in vitro studies have investigated the effects of seminal plasma on endometrium, however, the importance of this study compared to the in vitro investigations, is that the in vitro studies ignore the effect of female hormones on sperm and endometrial interaction (19), (25-27). The results of various studies have shown that sperm-epithelial cell interaction creates a successful embryo implantation in three different ways.

The first theory is to achieve a successful fertilization. Sperm should be capacitated in the female reproductive tract, necessitating a sperm interaction with the female epithelial cells (28). The first prerequisite for pregnancy in humans and other mammals is fertilization; to achieve this, the sperm capacitation in the female reproductive tract is completed, whereas this capacitation is initiated by the interaction of the sperm with the cervix (29). A study conducted by Reeve and Ledger showed that the Arg-Gly-Asp (RGD) sequence in the epithelial cell of endometrium makes a better interaction between the sperm and the endometrium (30). This interaction can be one of the important factors in the embryo implantation (31). The movement of the sperm in the uterine epithelial cell leads to the expression of some factors from secretory cells that provide the conditions and nutrients for embryo implantation (32), sperm-endometrium interaction cause the ATP secretion from the epithelial cell of the endometrium, an essential factor for the capacitation and the movement of the cell to the fallopian tube (33).

The second theory is that an interaction between sperm and the endometrium leads to the expression of inflammatory factors involved in the implantation pathway (32, 34). Kaczmarek and colleague showed that seminal plasma is effective in expressing endometrial cytokines in rabbits, and had been led to the expression of *EGF*, *VEGF*, and *FGF2* (35). In addition, Gutsche and colleague confirmed that seminal plasma induces the expression of cytokines and growth factors such as *LIF and VEGF* in the endometrium (36). Previous studies have shown that the passage of seminal plasma from endometrium lead to genomic alteration, such as the expression of *FGF* (37), which is consistent with the present study. Carp and colleague showed a higher implantation rate in mice exposed to semen after embryo transfer in their endometrium (38). The *VEGF*, *FGF2*, and *EGF* increase endometrial angiogenesis and subsequently lead to a successful implantation (22). The *LIF* is also one of the cytokines that plays a role in the pathway of proliferation, differentiation, and decidualization (21). Artificial insemination in sheep also benefits from cervical spermatozoa exposure and results in an increased percentage of pregnant ewes (39). As shown in Bellinge and colleague's study, exposing the uterus to sperm during the oocyte retrieval in an IVF cycle increases the implantation rate (40). However, clinical studies have shown that exposure to semen in ART during embryo transfer increases the embryo implantation rate, but the mechanism of this effect is yet unknown.

The third theory is that the interaction between seminal plasma and the endometrium is effective on steroid hormones, prostaglandins, and peptide hormones (41). As mentioned earlier, the sperm-endometrium interaction causes the secretion of inflammatory factors that affect the steroid hormones and prostaglandins (42). Steroid hormones play a role in the secretion of inflammatory factors involved in innate immunity, including cytokines (43). Cytokines play an important role in endometrial receptivity, which is consistent with the second theory. In this study, the expression of genes involved in the pathway of cytokines such as *LIF* was increased.

Moreover, studies about the sperm and seminal plasma-endometrium interaction are not sufficient and this is an open issue. Therefore, we tried to provide information about the potential effects of sperm and seminal plasma-uterine epithelium interaction in the genes involved in endometrial receptivity-related pathways. The results of this study have shown its use in IVF. The study also examines the human cellular level, determines the appropriate dose and volume of sperm seminal fluid to influence embryo implantation, and can be used for uterine preparation in IVF cycles.

According to the results of the present study, it can be concluded that seminal plasma regulates the *LIF*, *LIFR*, *HOXA10*, *MUC1*, *PGR*, *CSF*, *VEGF*, *HBEGF*, *EGF*, and *FGF2* expression in the mouse endometrium in vivo model, and because these genes play an important role in implantation and can increase the rate of implantation by facilitating the receptivity of the endometrium. It should be noted that in patients undergoing IVF cycles, the seminal plasma cannot be contacted with endometrium and patients will avoid intercourse before and after taking the ovum. It can be considered that implantation failures in these patients can be attributed to the lack of the stimulation of endometrial cells from seminal plasma.

## 5. Conclusion

Finally, it was found that the seminal plasma is effective in expressing the involved genes in implantation in the in vivo model. In fact, seminal plasma increases endometrial receptivity prior to an embryo transfer. Seminal plasma induces the expression of *LIF, LIFR, MUC1, VEGF, EGF,* and *FGF2* genes that increase stromal cell survival and endometrial tissue proliferation before an implantation. According to the obtained results in this study, seminal plasma can be introduced as an auxiliary and effective factor in successful implantation. Taken together, our findings and those of others suggest that the effects of seminal plasma on the endometrium and pregnancy should be investigated in an in vivo/in vitro model for confirmation of these results.

##  Conflict of Interest

The authors declare that there is no competing interest.

## References

[1] Özatika O, Mungan T, Dag I, Musmuld A. The effect of sperm activation on pinopod formation in endometrial epithelium. *J Anat Soc India* 2016; 65: S5–S10.

[2] Rincón A, Bolumar D, Valbuena D, Simón C. Use of Molecular markers of endometrial receptivity. In: Rizk B, Khalaf Y. Controversies in assisted reproduction. USA: CRC Press; 2020.

[3] Neykova K, Tosto V, Giardina I, Tsibizova V, Vakrilov G. Endometrial receptivity and pregnancy outcome. *J Matern Fetal Neonatal Med* 2020; 2: 1–15. 10.1080/14767058.2020.1787977 32744104

[4] Ajdary M, Keyhanfar F, Aflatoonian R, Amani A, Amjadi F, Zandieh Z, et al. Design and evaluation of a novel nanodrug delivery system for reducing the side effects of clomiphene citrate on endometrium. *DARU J Pharm Sci* 2020; 28: 1–10. 10.1007/s40199-019-00310-2 PMC7704853 32483681

[5] Ajdary M, Farzan S, Razavi Y, Arabdolatabadi A, Haghparast A. Effects of morphine on serum reproductive hormone levels and the expression of genes involved in fertility-related pathways in male rats. *Iran J Pharm Res* 2019; 19: 1–26. 10.22037/ijpr.2019.112119.13544 PMC8170771 34400949

[6] Lee SK, Kim CJ, Kim DJ, Kang JH. Immune cells in the female reproductive tract. *Immune Netw* 2015; 15: 16–26. 10.4110/in.2015.15.1.16 PMC4338264 25713505

[7] Ibrahim LA, Rizo JA, Fontes PLP, Lamb GC, Bromfield JJ. Seminal plasma modulates expression of endometrial inflammatory meditators in the bovine. *Biol Reprod* 2019; 100: 660–671. 10.1093/biolre/ioy226 PMC6437260 30329018

[8] Berger C, Boggavarapu NR, Menezes J, Lalitkumar PGL, Gemzell-Danielsson K. Effects of ulipristal acetate on human embryo attachment and endometrial cell gene expression in an in vitro co-culture system. *Hum Reprod *2015; 30: 800–811. 10.1093/humrep/dev030 25740886

[9] Robertson SA. Seminal plasma and male factor signalling in the female reproductive tract. *Cell Tissue Res *2005; 322: 43–52. 10.1007/s00441-005-1127-3 15909166

[10] He B, Ni Zl, Kong SB, Lu JH, Wang HB. Homeobox genes for embryo implantation: From mouse to human. *Anim Model Exp Med* 2018; 1: 14–22. 10.1002/ame2.12002 PMC6357426 30891542

[11] Namiki T, Ito J, Kashiwazaki N. Molecular mechanisms of embryonic implantation in mammals: Lessons from the gene manipulation of mice. *Reprod Med Biol* 2018; 17: 331–342. 10.1002/rmb2.12103 PMC6194304 30377389

[12] Yuan J, Deng W, Cha J, Sun X, Borg JP, Dey SK. Tridimensional visualization reveals direct communication between the embryo and glands critical for implantation. *Nat Commun* 2018; 9: 603–615. 10.1038/s41467-018-03092-4 PMC5807548 29426931

[13] Ribatti D, Tamma R. The chick embryo chorioallantoic membrane as an in vivo experimental model to study human neuroblastoma. *J Cell Physiol *2018; 234: 152–157. 10.1002/jcp.26773 30078179

[14] Fermin LM, Pain SJ, Morel PCH, Gedye KR, Kenyon PR, Blair HT. Effect of exogenous progesterone on embryo size and ewe uterine gene expression in an ovine `dam size'model of maternal constraint. *Reprod Fertil Dev* 2018; 30: 766–778. 10.1071/RD17096 29157356

[15] Camargo-Díaz F, García V, Ocampo-Bárcenas A, González-Marquez H, López-Bayghen E. Colony stimulating factor-1 and leukemia inhibitor factor expression from current-cycle cannula isolated endometrial cells are associated with increased endometrial receptivity and pregnancy. *BMC Women's Health* 2017; 17: 63–69. 10.1186/s12905-017-0418-7 PMC5567912 28830391

[16] Miah AG, Salma U, Hamano K, Schellander K. Physiological roles of relaxin in prefertilizing activities of spermatozoa. *Anim Reprod Sci* 2015; 161: 1–15. 10.1016/j.anireprosci.2015.07.013 26300501

[17] Artimani T, Karimi J, Mehdizadeh M, Yavangi M, Khanlarzadeh E, Ghorbani M, et al. Evaluation of pro-oxidant-antioxidant balance (PAB) and its association with inflammatory cytokines in polycystic ovary syndrome (PCOS). *Gynecol Endocrinol *2018; 34: 148–152. 10.1080/09513590.2017.1371691 28868943

[18] Chelongar R, Hajihosseinlo A, Ajdary M. The effect of Igf-1 and pit-1 genes polymorphisms on fat-tail measurements (fat-tail dimensions) in Makooei sheep. *Adv Environ Biol* 2014; 8: 862–868.

[19] Robertson SA, Ingman WV, O'Leary S, Sharkey DJ, Tremellen KP. Transforming growth factor β-a mediator of immune deviation in seminal plasma. *J Reprod Immunol* 2002; 57: 109–128. 10.1016/s0165-0378(02)00015-3 12385837

[20] Cheng J, Rosario G, Cohen TV, Hu J, Stewart CL. Tissue-specific ablation of the LIF receptor in the murine uterine epithelium results in implantation failure. *Endocrinology *2017; 158: 1916–1928. 10.1210/en.2017-00103 PMC5460932 28368537

[21] Chen J, Zhao X, Ao L, Yin T, Yang J. Transcriptomic changes and potential regulatory mechanism of intrauterine human chorionic gonadotropin co-cultured with peripheral blood mononuclear cells infusion in mice with embryonic implantation dysfunction. *Ann Transl Med* 2020; 8: 99–113. 10.21037/atm.2019.12.109 PMC7049043 32175392

[22] Shim SH, Kim JO, Jeon YJ, An HJ, Lee HA, Kim JH, et al. Association between vascular endothelial growth factor promoter polymorphisms and the risk of recurrent implantation failure. *Exp Ther Med* 2018; 15: 2109–2119. 10.3892/etm.2017.5641 PMC5776554 29434813

[23] Yue L, Yu HF, Yang ZQ, Tian XC, Zheng LW, Guo B. Egr2 mediates the differentiation of mouse uterine stromal cells responsiveness to HB-EGF during decidualization. *J Exp Zoo B Mol Dev Evol *2018; 330: 215–224. 10.1002/jez.b.22807 29781132

[24] Hu M, Zhang Y, Feng J, Xu X, Zhang J, Zhao W, et al. Uterine progesterone signaling is a target for metformin therapy in PCOS-like rats. *J Endocrinol* 2018; 237: 123–137. 10.1530/JOE-18-0086 29535146

[25] Altmäe S, Martinez-Conejero JA, Salumets A, Simon C, Horcajadas JA, Stavreus-Evers A. Endometrial gene expression analysis at the time of embryo implantation in women with unexplained infertility. *Mol Hum Reprod* 2010; 16: 178–187. 10.1093/molehr/gap102 19933690

[26] Hamlett WC, Musick JA, Hysell CK, Sever DM. Uterine epithelial-sperm interaction, endometrial cycle and sperm storage in the terminal zone of the oviducal gland in the placental smoothhound, Mustelus canis. *J Exp Zool *2002; 292: 129–144. 10.1002/jez.1149 11754029

[27] Zandieh Z, Ashrafi M, Aflatoonian K, Aflatoonian R. Human sperm DNA damage has an effect on immunological interaction between spermatozoa and fallopian tube. *Andrology *2019; 7: 228–234. 10.1111/andr.12574 30663256

[28] Robertson SA, O'Leary S, Armstrong DT. Influence of semen on inflammatory modulators of embryo implantation. *Soc Reprod Fertil Suppl *2006; 62: 231–245. 16866321

[29] Elweza AE, Ezz MA, Acosta TJ, Talukder AK, Shimizu T, Hayakawa H, et al. A proinflammatory response of bovine endometrial epithelial cells to active sperm in vitro. *Mol Reprod Dev* 2018; 85: 215–226. 10.1002/mrd.22955 29337420

[30] Reeve L, Ledger WL, Pacey AA. Does the Arg-Gly-Asp (RGD) adhesion sequence play a role in mediating sperm interaction with the human endosalpinx? *Hum Reprod *2003; 18: 1461–1468. 10.1093/humrep/deg296 12832373

[31] Gomes GM, Crespilho AM, Leão KM, Jacob JCF, Gomes LPM, Segabinazzi LG, et al. Can sperm selection, inseminating dose, and artificial insemination technique influence endometrial inflammatory response in mares? *J Equine Vet Sci* 2019; 73: 43–47.

[32] Bahar L, Kahraman S, Akkuş M, Baykal T. Fine structure and immunohistochemical evaluation of endometrium in fertile and infertile women with implantation failure. *Dicle Tıp Dergisi* 2012; 39: 269–275.

[33] Burnstock G. Purinergic signalling in the reproductive system in health and disease. *Purinergic Signal* 2014; 10: 157–187. 10.1007/s11302-013-9399-7 PMC3944041 24271059

[34] Cakmak H, Taylor HS. Implantation failure: molecular mechanisms and clinical treatment. *Hum Reprod Update* 2011; 17: 242–253. 10.1093/humupd/dmq037 PMC3039220 20729534

[35] Kaczmarek MM, Krawczynski K, Blitek A, Kiewisz J, Schams D, Ziecik AJ. Seminal plasma affects prostaglandin synthesis in the porcine oviduct. *Theriogenology* 2010; 74: 1207–1220. 10.1016/j.theriogenology.2010.05.024 20615530

[36] Gutsche S, Von Wolff M, Strowitzki T, Thaler CJ. Seminal plasma induces mRNA expression of IL-1beta, IL-6 and LIF in endometrial epithelial cells in vitro. *Mol Hum Reprod* 2003; 9: 785–791. 10.1093/molehr/gag095 14614040

[37] Alvarez-Rodriguez M, Atikuzzaman M, Venhoranta H, Wright D, Rodriguez-Martinez H. Expression of immune regulatory genes in the porcine internal genital tract is differentially triggered by spermatozoa and seminal plasma. *Int J Mol Sci *2019; 20: 513–532. 10.3390/ijms20030513 PMC6387272 30691059

[38] Carp HJ, Serr DM, Mashiach S, Nebel L. Influence of insemination on the implantation of transferred rat blastocysts. *Gynecol Obstet Invest *1984; 18: 194–198. 10.1159/000299080 6510779

[39] Maxwell WM, Evans G, Mortimer ST, Gillan L, Gellatly ES, McPhie CA. Normal fertility in ewes after cervical insemination with frozen-thawed spermatozoa supplemented with seminal plasma. *Reprod Fertil Dev* 1999; 11: 123–126. 10.1071/rd99046 10735556

[40] Bellinge BS, Copeland CM, Thomas TD, Mazzucchelli RE, O'Neil G, Cohen MJ. The influence of patient insemination on the implantation rate in an in vitro fertilization and embryo transfer program. *Fertil Steril *1986; 46: 252–256. 10.1016/s0015-0282(16)49521-x 3732531

[41] Cicinelli E, De Ziegler D. Transvaginal progesterone: evidence for a new functional'portal system'flowing from the vagina to the uterus. *Hum Reprod Update* 1999; 5: 365–372. 10.1093/humupd/5.4.365 10465526

[42] Pelzer ES, Huygens F, Beagley KW. Steroid hormone dependent inflammation and regulation in the endometrium in women with dysfunctional menstrual cycles: Is there a role for toll-like receptor activation via PAMPs and DAMPs? *J Microb Biochem Technol *2016; 8: 344–357.

[43] Wira CR, Fahey JV, Sentman CL, Pioli PA, Shen L. Innate and adaptive immunity in female genital tract: cellular responses and interactions. *Immunol Rev *2005; 206: 306–335. 10.1111/j.0105-2896.2005.00287.x 16048557

